# Chemotherapy resumption in breast cancer patient after COVID-19

**DOI:** 10.1186/s40792-021-01253-0

**Published:** 2021-07-21

**Authors:** Julian Horiguchi, Ayako Nakashoji, Naoki Kawahara, Akira Matsui, Takayuki Kinoshita

**Affiliations:** 1 Department of Surgery, National Hospital Organization Tokyo Medical Center, 2-5-1, Higashigaoka, Setagaya-ku, Tokyo, 152-8902 Japan; 2 Department of Breast Surgery, National Hospital Organization Tokyo Medical Center, 2-5-1, Higashigaoka, Setagaya-ku, Tokyo, 152-8902 Japan

**Keywords:** COVID-19, Severe acute respiratory syndrome coronavirus 2, Resumption, Breast cancer, Chemotherapy, Case report

## Abstract

**Background:**

While many studies have verified the effect of recent anti-cancer treatment in patients with COVID-19, there are no data on the optimal time for cancer treatment resumption, as well as the safety of chemotherapy in COVID-19 patients. As many cancer patients are recovering from COVID-19, there is an urgent need for reliable clinical information. Herein, we report a case of invasive ductal carcinoma in which we were able to successfully resume chemotherapy after infection with SAR-CoV-2.

**Case presentation:**

The patient was a 38-year-old non-smoking Japanese woman with no significant medical history. She had fever on days 5 and 6 of her second course of adjuvant FEC therapy, and on day 7, she tested positive for SARS-CoV-2 by RT-PCR. She was hospitalized for 11 days. We resumed the therapy on day 25 after discharge, as she had no remaining clinical symptoms. The patient completed four courses of the initial chemotherapy without any major adverse events nor the recurrence of COVID-19, and subsequently completed four courses of docetaxel as her second regimen therapy.

**Conclusions:**

Evaluating the risk for each patient is essential when resuming anti-cancer therapy in cancer patient’s post-COVID-19.

## Background

The outbreak of severe acute respiratory syndrome coronavirus 2 (SARS-CoV-2)-associated disease (COVID-19) has led to a global pandemic. It has imposed significant challenges in the management of chronic illnesses, including cancer. Some reports suggest that cancer patients could be at an increased risk of developing COVID-19-related complications, and many oncological societies have issued guidelines to assist clinical decisions and management during this crisis [1, 2]. Many studies have verified the effect of recent anti-cancer treatment in patients with COVID-19; however, the results remain controversial [3, 4]. Moreover, no study has evaluated the risk for post-COVID-19 patients who resumed their anti-cancer treatment. Only a few case reports exist on this issue, and one report revealed a severe reactivation of the virus after the resumption of treatment [5–7]. Furthermore, the aforementioned guidelines do not include instructions regarding the optimal time for cancer treatment resumption, as well as the safety of chemotherapy resumption. As many cancer patients are recovering from COVID-19, there is an urgent need for reliable clinical information.

Breast cancer is the most common malignancy in women around the world, and also one of the malignancies in which a wide variety of anti-cancer medicine is indispensable for treatment. Herein, we report a case of invasive ductal carcinoma in which we were able to resume chemotherapy after infection with SAR-CoV-2.

## Case presentation

The patient was a 38-year-old non-smoking Japanese woman with no significant medical history. She was diagnosed with invasive ductal carcinoma of the right breast, and in August 2020, we performed partial right breast resection and axillary lymph node dissection. Her post-operative diagnosis was pT2N2aM0 pStage IIB, ER(+) PgR(+) HER2(−), and she was administered her first course of chemotherapy with (5-Fluorouracil 500 mg/m^2^), epirubicin hydrochloride (100 mg/m^2^), and cyclophosphamide (500 mg/m^2^) (FEC) every 3 weeks in mid-September.

A day before receiving the second course of chemotherapy, she had a fever of 38.0 °C. The next morning, her fever had alleviated, and she visited our outpatient department. She had no cough at this point. Her blood test showed no abnormalities; therefore, we administered a second course of chemotherapy. As an antiemetic agent, she received 8 mg/day of dexamethasone on days 1–4 and an aprepitant on days 1–3. According to our hospital’s standardized treatment, on day 3, she received a shot of peg-filgrastim (3.6 mg).

On days 5 and 6 after the administration of chemotherapy, she had a fever ranging from 39–40 °C. On day 7, she tested positive for SARS-CoV-2 by reverse transcription polymerase chain reaction (RT-PCR).

**Fig. 1:**
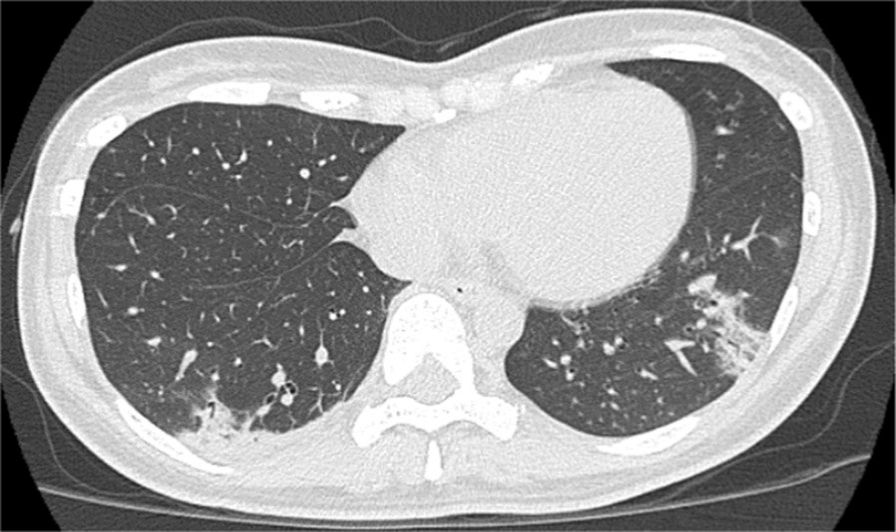
Chest CT findings. Chest CT findings at the diagnosis of COVID-19 showing ground-glass opacity in the peripheral lesion of both lungs

**Fig. 2:**
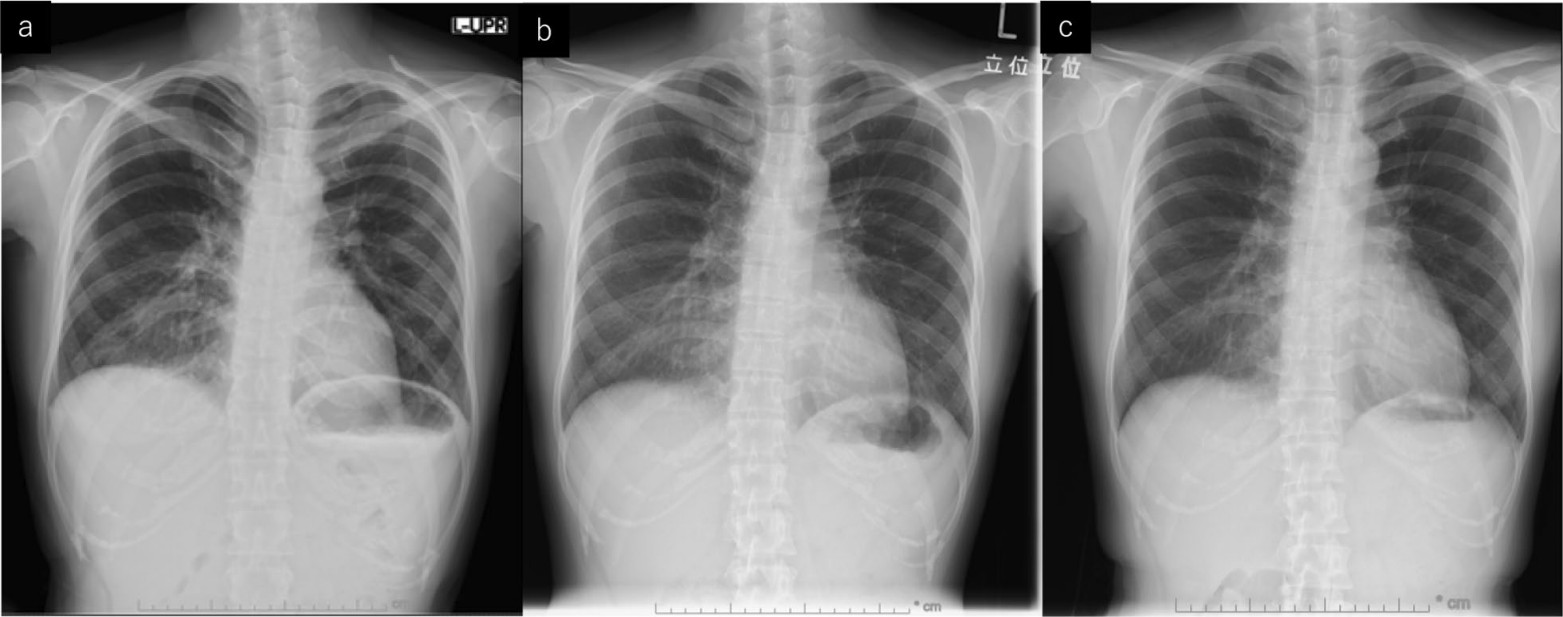
Chest X-ray findings. **a** X-ray on day of COVID-19 diagnosis indicating infiltrative shadows in the peripheral lesion of both lungs. **b** X-ray after 2 weeks from discharge showing benign organized pneumonia in both lungs. **c** X-ray 3 months after discharge showing the dissipation of organized pneumonia

The next day, she was admitted to our hospital for treatment. Her main symptoms were cough and high temperature. Additional symptoms included fatigue and mild joint pain. She did not show any signs of hypoxemia. Laboratory tests revealed the following findings: white blood cell count, 1600/μL with 77.0% neutrophils, 17.0% lymphocytes, 3.0% monocytes, 1.0% eosinophils, 2.0% basophils; hemoglobin, 12.6 g/dL; platelet count, 140,000/μL; and C-reactive protein 3.62 mg/dL. Chest computed tomography (CT) revealed ground-glass opacity in the peripheral lesions of both lungs (Fig. 1). Chest X-ray indicated infiltrative shadows in the peripheral lesions of both lungs (Fig. 2a). She was diagnosed with COVID-19 with grade three leukopenia and grade two neutropenia. Antibiotic therapy (levofloxacin 500 mg every 24 h, taken orally) was administered after collecting blood cultures.

**Fig. 3:**
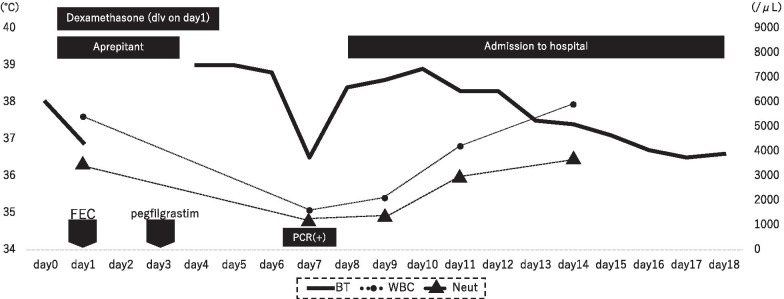
Timeline before and after COVID-19 diagnosis. *BT* body temperature, *WBC* white blood cell count, *neutro* neutrophil count, *FEC* 5-fluorouracil, epirubicin hydrochloride, and cyclophosphamide

Antibiotic therapy was discontinued after a blood test showed normalization of her white blood cell count on day 5 after the diagnosis. Intermittent cough and high temperature persisted until day 8 after the diagnosis. After confirming that at least 72 h had passed, since her last episode of fever, she was discharged 12 days after the diagnosis (day 19 after the administration of chemotherapy Fig. 3).

The patient visited our outpatient department 2 weeks after discharge for a chest X-ray and blood test follow-up. The blood test indicated the normalization of inflammatory markers, and the chest X-ray images showed benign organized pneumonia in the peripheral lesion of both lungs, matching the ground-glass opacities seen in the CT before (Fig. 2b).

In considering resumption of chemotherapy, we consulted a pulmonologist. Since no criteria exist, we were advised based on clinical experience, to defer resumption until at least 2 weeks had passed after discharge. Taking this into account, we decided to resume anti-cancer therapy after adequately informing the patient about the risks and benefits. FEC therapy every 3 weeks was resumed on day 25 after discharge (day 43 after the last administration of the chemotherapy). The patient was able to complete four courses of the initial chemotherapy without any major adverse events, and we were able to institute docetaxel (70 mg/m^2^) every 3 weeks as the second regimen of chemotherapy. Organized pneumonia was eclipsed in the follow-up chest X-ray image taken at the beginning of docetaxel therapy (Fig. 2c). She completed four courses of docetaxel without any major adverse events.

## Discussion

To the best of our knowledge, this is the first case report of a breast cancer patient who was successfully treated with chemotherapy after a COVID-19 diagnosis.

Patients with cancer during their course of cytotoxic chemotherapy are affected by bacterial and viral infections; in the case of SARS-CoV-2 infection, there have been retrospective studies to verify the effect of recent cancer treatment on the COVID-19 course; however, the results have been controversial [3, 8–10]. Moreover, although there have been many recommendations for the prioritization and treatment of cancer patients during the COVID‑19 pandemic, there are no criteria concerning the resumption of chemotherapy after COVID-19 diagnosis. Furthermore, no study has evaluated the risk for post-COVID-19 patients who resumed anti-cancer treatment. However, long discontinuation of chemotherapy is undesirable, considering the dose intensity of the therapy. Although the risk of immediate disease progression may not be large in the adjuvant setting, as in this case, permanent discontinuation of the therapy would certainly be disadvantageous for patient prognosis from the viewpoint of cancer recurrence. Reliable information for safe resumption of chemotherapy in post-COVID-19 patients is strongly warranted.

This case report demonstrates two important clinical issues. First, our findings suggest that the resumption of chemotherapy can be considered after COVID-19 symptoms have disappeared, even if the patients have residual radiological findings in the lungs. Our patient had experienced ground-glass opacity in the peripheral lesion of both lungs in the chest CT images taken at the time of administration; the post-discharge follow-up chest X-ray images showed benign organized pneumonia, although the patient had no clinical symptoms or COVID-19-related complications at this point. Subsequently, the patient did not experience any pulmonary adverse events after the resumption of chemotherapy. It has been reported that a majority of COVID-19 patients still have mild-to-substantial residual radiological lung abnormalities post-recovery [11]. Lung organization is a common and nearly universal response to lung injury, and although most of them heal without permanent injury, some patients are at risk of developing fibrosis, which can be severe [12]. However, a study reported that most COVID-19 patients presenting organizing pneumonia cases had good prognosis with only 2.8% resulting in major adverse events [13]. Thus, the resumption of chemotherapy may not necessarily have to be delayed, even when patients show residual radiological findings.

Second, evaluating the risk for each patient is essential when resuming anti-cancer therapy in cancer patients post-COVID-19, taking into account that cancer patients may be at a higher risk of re-infection due to a lower detection rate of SARS-CoV-2 antibodies post-infection [14]. Previous reports have attributed the risks of COVID-19 to pre-existing comorbidities, the type of tumor, and anti-cancer treatment in cancer patients. With regard to comorbidities, diabetes, hypertension, and cardiovascular diseases are predisposed to poor clinical outcomes in patients with COVID-19 [15]. A cohort study on COVID-19 in breast cancer patients showed that hypertension and age (> 70 years) were associated with a higher risk of intensive care unit admission, death, or both [16]. With respect to tumor type, chemotherapy for patients with hematological malignancies has been reported to lead to higher mortality in COVID-19 [4]. Furthermore, there has been a case report describing severe COVID-19 virus reactivation following the resumption of chemotherapy for B cell acute lymphoblastic leukemia [7]. Considering the treatment type, the risk of pneumonitis cannot be ignored in patients receiving molecular target drugs. Some clinical symptoms and radiological findings of pneumonitis can be attributed to COVID-19 as well as immune-related adverse events. Identifying the exact cause and promptly initiating the most appropriate treatment may be challenging during the COVID-19 pandemic [17]; therefore, the resumption of molecular target drugs may be considered a higher risk than other anti-cancer treatments. In this case, the patient was a 38-year-old woman with no significant medical history and was receiving cytotoxic chemotherapy for a solid tumor. Thus, the risk of resuming chemotherapy was perceived to be relatively low.

However, some questions remain. Although lymphopenia at COVID-19 diagnosis has been reported to be associated with worse outcomes [3], our patient displayed only mild symptoms with a lymphopenia peri-COVID-19 diagnosis. This may have been because the cause of lymphopenia was mainly attributed to the chemotherapy prior to infection and not the infection itself; however, the causal relationship could not be determined. In addition, considering that dexamethasone is effective for COVID-19 treatment [18], there is a possibility that the dexamethasone used after the administration of chemotherapy and prior to COVID-19 diagnosis may correlate with mild COVID-19 symptoms; however, this hypothesis remains open to debate.

In conclusion, our report suggests that it may be feasible to resume chemotherapy in low-risk cancer patients with COVID-19 after all symptoms have resolved. We believe that this is an important preliminary information for the management of COVID-19 in cancer patients and may help oncologists make clinical decisions in the era of the COVID-19 pandemic. Further accumulation of data and studies is required to confirm our conclusions.

## Data Availability

The data that support the findings of this study are available from the corresponding author upon reasonable request.

## Abbreviations

SARS-CoV-2: Severe acute respiratory syndrome coronavirus 2
COVID-19: Severe acute respiratory syndrome coronavirus 2 associated disease
FEC: Chemotherapy with (5-Fluorouracil 500 mg/m^2^), epirubicin hydrochloride (100 mg/m^2^), and cyclophosphamide (500 mg/m^2^)
RT-PCR: Reverse transcription polymerase chain reaction
CT: Chest computed tomography

## Declarations

### Research involving human and/or animals rights

This article does not contain any studies with human participants performed by any of the authors.
